# Diagnostic accuracy of AI-assisted chest radiographs in tuberculosis screening: A Ghanaian clinical study

**DOI:** 10.1371/journal.pone.0342988

**Published:** 2026-03-27

**Authors:** Derick Seyram Sule, Kofi Adesi Kyei, William Kwadwo Antwi, Godwill Acquah, Klenam Dzefi-Tettey, Joseph Daniels, Andrew Yaw Nyantakyi

**Affiliations:** 1 Department of Radiography, School of Biomedical and Allied Health Sciences (SBAHS), University of Ghana, Accra, Ghana; 2 National Radiotherapy, Oncology and Nuclear Medicine Center (NRONMC), Korle Bu Teaching Hospital, Accra, Ghana; 3 Department of Radiology, Korle Bu Teaching Hospital (KBTH), Accra, Ghana; 4 Department of Radiology, School of Medicine, University of Health and Allied Sciences, Ho, Ghana; 5 Department of Oncology, Cape Coast Teaching Hospital (CCTH), Cape Coast, Ghana; Gulu University, UGANDA

## Abstract

**Background:**

Tuberculosis remains a major global health challenge, particularly in resource-limited settings where access to expert radiological interpretation is constrained. Artificial intelligence offers a promising solution to enhance diagnostic accuracy and efficiency in TB screening.

**Aim:**

This study aimed to evaluate the diagnostic performance of an AI-based system compared to a radiologist in screening for TB using chest X-rays from 1,010 patients.

**Methods:**

Patients were adults ≥18 years with suspected TB in a high-burden setting. GeneXpert MTB/RIF served as reference to assess accuracy, sensitivity, specificity, PPV, NPV, and AUC for radiologist and AI TB predictions. Comparisons used McNemar’s test and Cohen’s kappa to evaluate agreement and significance of differences.

**Results:**

The AI system demonstrated superior performance with an accuracy of 91%, sensitivity of 86%, specificity of 93%, PPV of 85%, NPV of 94%, and AUC of 0.90. In contrast, the radiologist achieved an accuracy of 86%, sensitivity of 84%, specificity of 87%, PPV of 76%, NPV of 92%, and AUC of 0.86. McNemar’s test revealed a statistically significant difference between the two modalities (p = 0.0021). Cohen’s kappa indicated substantial agreement between AI and GeneXpert MTB/RIF result (κ = 0.79), moderate agreement for the radiologist and GeneXpert MTB/RIF result (κ = 0.69), and moderate agreement between radiologist and AI predictions (κ = 0.53).

**Conclusion:**

The AI system outperformed the radiologist in TB screening, demonstrating higher diagnostic accuracy and agreement with GeneXpert MTB/RIF result. These findings support the integration of AI into TB screening workflows, particularly in settings with limited access to expert radiological interpretation.

## Introduction

Tuberculosis (TB) remains a significant public health challenge globally, particularly in low- and middle-income countries (LMICs) including Ghana [1]. Despite advances in treatment and control strategies, early detection continues to be a bottleneck in TB elimination efforts [2,3]. A Two-Dimensional Chest radiography (2D CXR) is a widely used screening tool due to its accessibility and cost-effectiveness, yet its diagnostic accuracy is often limited by inter-reader variability and limited trained radiologists [4,5].

Advancement in Artificial Intelligence (AI) has introduced promising solutions to enhance the interpretation of chest radiographs. AI-assisted diagnostic tools, particularly those leveraging deep learning algorithms, have demonstrated potential in automating the detection of pulmonary abnormalities consistent with features TB [6–8]. Combining AI with human readers improves diagnostic accuracy. AI-assisted chest radiograph interpretation has gained traction as a supplementary tool in TB screening, with studies reporting as high 92−94% sensitivity and specificity of 98.2−95% [7,9]. These findings underscore the potential of AI to support large-scale screening programs, particularly in LMICs where there is limited radiologist.

Despite these advances, challenges remain. Santosh, Shen, and Zhang (2022) provided an overview of deep learning approaches for TB screening, noting that algorithm performance is influenced by image quality, dataset diversity and training methodology [10]. A further review emphasized the need for contextual validation and integration into clinical workflows [11]. Furthermore, concerns about false positives persist hence the need for robust validation in diverse population. In light of this, there has been increased investment in digital health tools, including AI, to accelerate TB elimination efforts [12,13]. The applicability of AI tools in African contexts is still emerging. Recent innovations have focused on deploying AI tools in real-world, resource-constrained settings with promising results [14,15]. These tools offer rapid, scalable and standardized assessments, which are especially valuable in resource-constrained settings.

In Ghana, the TB case detection rate has decreased [16]. The integration of AI into diagnostic workflows could significantly reduce time to diagnosis and improve screening outcomes in this high prevalent TB region with unevenly distributed radiological expertise [17]. Only a few studies have systematically assessed the diagnostic accuracy, the clinical utility and diagnostic accuracy of these AI-assisted radiography for TB tools in the Ghanaian context remain underexplored. This gap highlights the need for Ghanaian context-specific research to inform policy and clinical practice. This study seeks to bridge the evidence gap and support informed implementation of AI in TB screening by evaluating the performance of AI-assisted 2D CXR interpretation in TB screening compared to radiologist within a Ghanaian clinical setting.

## Methods

The study employed a descriptive, retrospective, quantitative cross-sectional design and was conducted at the Family Medicine department of the Korle Bu Teaching Hospital. The department is a major point of entry to the facility and offers a range of basic and limited specialized care. At this department there is a dedicated X-ray unit. The study period spanned from 1^st^ January 2021–1^st^ January 2025, and aimed to assess the performance of a Convolutional Neural Network (CNN)-assisted model in detecting pulmonary tuberculosis (TB) using 2D CXR. Data collection started on 30^th^ March,2024 upon receipt of ethical approval. A total sampling technique was employed during the study period using a consecutive approach. Patients included into the study were >18 years old with microbiological confirmation of TB via GeneXpert MTB/RIF assay results, as recorded by attending physicians in the hospital’s health information management system. Radiographic images taken were posterior-anterior (PA). All radiographs were exported in DICOM format and converted to PNG. Images were resized to 224 × 224 pixels and normalized to enhance contrast and mitigate illumination variability. A ResNet50-based CNN architecture was employed, initialized with weights from the ImageNet dataset and fine-tuned using a combined dataset of publicly available bacteriologically confirmed TB radiographs from the same facility from preceeding years. The CNN model was independently applied to the set of chest radiographs, generating probability scores for tuberculosis (TB) presence.

A classification threshold of 0.5 was used to determine TB-positive and TB-negative predictions. Data augmentation techniques including horizontal flipping, zooming, and rotation were applied to increase model robustness. Radiographic interpretation was based on a retrospective review conducted by certified radiologist with experience in thoracic imaging. The radiologist’s blinded original diagnostic reports documented at the time of clinical care were extracted from the hospital’s radiology information system without modification. These reports served as the human comparator for evaluating the CNN model’s performance. Model performance and radiologist interpretation were both compared against laboratory confirmation for sensitivity, specificity, accuracy, and area under the receiver operating characteristic curve (AUC-ROC). Discrepancies between the CNN model and radiologist reports were analyzed to identify patterns of diagnostic divergence.

**Fig 1:**
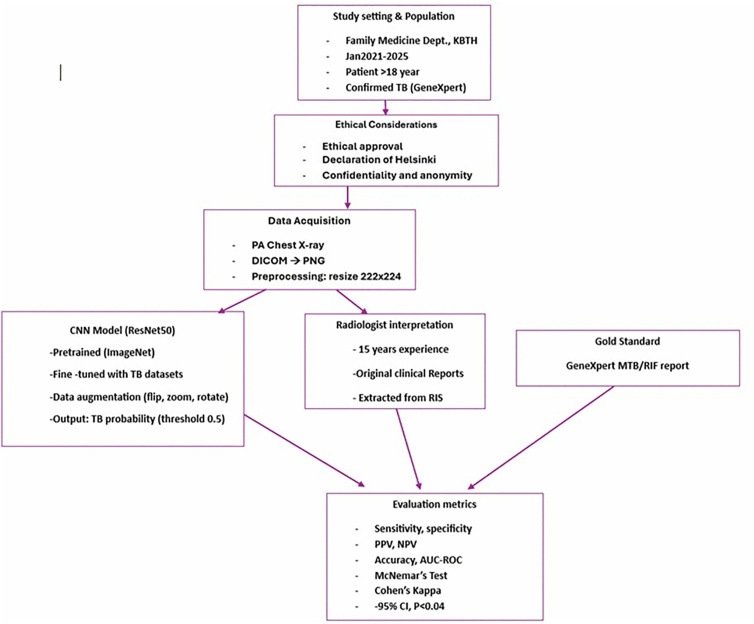
Framework of the study.

Statistical comparison was performed between model predictions and expert radiologist interpretations. McNemar’s test assessed differences in paired proportions, and Cohen’s kappa measured agreement. Confidence interval was computed at the 95% confidence level, with statistical significance set at *p* < 0.05, using SPSS v26. Ethical approval was obtained from the Ethics and Protocol Review Committee of the School of Biomedical and Allied Health Sciences, College of Health Sciences, University of Ghana. Secondary data was collected from patient hospital files hence consent to participate from patient was waived by ethical committee. Declaration of Helsinki, data confidentiality and patient anonymity were strictly adhered to and maintained throughout the study by removal of all patient identifiers and replacing them with unique study numbers. The framework of the study is depicted in Fig 1.

## Results

### Patient screening overview

A total of 1,010 patients that met the inclusion criteria were sampled during the period. The Gold standard of diagnosis in this study is laboratory confirmation of TB. In 323 patients, the test was positive and negative in 687 patients. Both radiologist positive imaging interpretations and AI prediction (357 and 327 patients respectively) were high compared to laboratory confirmation. However, the Ai prediction was closer to that of laboratory confirmation (327 versus 323 patients respectively) as compared to Radiologist review (357 versus 323 patients respectively). This depicted in Table 1. In the subgroup of patients deemed positive by Radiologist review, 323 were truly positive for TB by laboratory confirmation and the remaining 34 falsely positive (thus negative on laboratory confirmation. For the AI prediction, 301 and 26 patients were truly positive and falsely positive respectively on laboratory confirmation as shown in Table 2.

**Table 1:** Distribution of TB Status by Laboratory Confirmation, Radiologist, and AI Prediction.

Category	Positive	Negative
Laboratory confirmation	323	687
Radiologist	357	653
AI Prediction	327	683

**Table 2:** Radiologist and AI Detection Outcomes Stratified by GeneXpert MTB/RIF Results.

GeneXpert MTB/RIF result	Radiologist Positive	AI Positive
Negative	34	26
Positive	323	301

**Fig 2:**
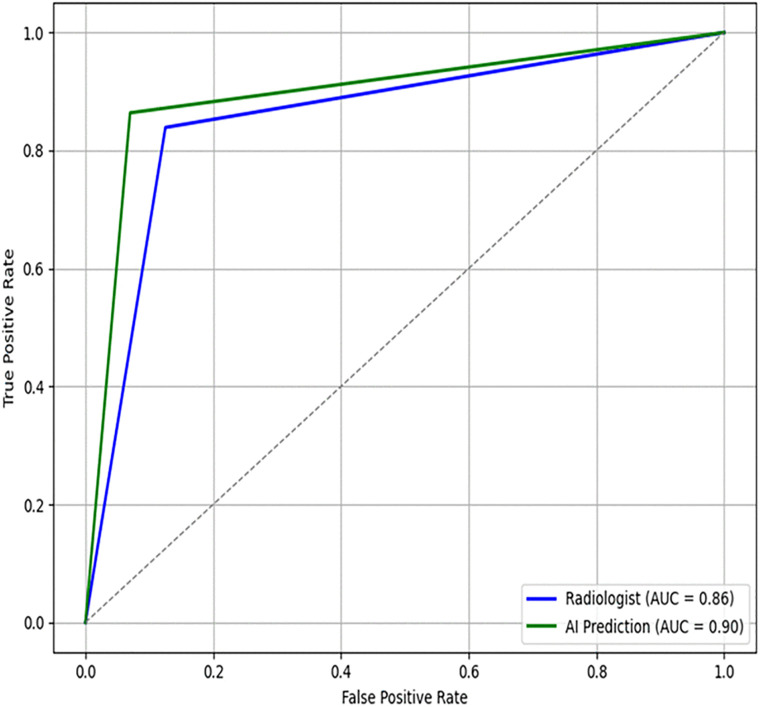
ROC Curve comparing radiologist performance and AI system.

The AI system shows a higher sensitivity (86%), specificity (93%) PPV (85%), NPV (94%) and AUC (90%) for AI prediction compared with that of radiologist, indicating superior classification ability in distinguishing TB-positive from TB-negative cases as shown in Table 3. The AI system shows a higher AUC, indicating superior classification ability in distinguishing TB-positive from TB-negative cases as depicted in Fig 2.

**Table 3:** Diagnostic Performance of Radiologist and AI Predictions Compared with GeneXpert MTB/RIF Reference Standard.

Metric	Radiologist	AI Prediction
Sensitivity	0.84	0.86
Specificity	0.87	0.93
PPV	0.76	0.85
NPV	0.92	0.94
AUC	0.86	0.90

### Statistical comparison using McNemar’s test

To evaluate the significance of differences McNemar’s test of significance of differences in paired proportions between radiologist and AI predictions showed a t- 9.46 with p = 0.0021

Calculated Cohen’s kappa to measure the level of agreement between the GeneXpert MTB/RIF result, radiologist, and AI predictions are shown in Table 4. The agreement between AI predictions and GeneXpert MTB/RIF result (κ = 0.79) is higher than that of the radiologist (κ = 0.69). Also, the agreement between radiologist and AI predictions was moderate (κ = 0.53).

**Table 4:** Cohen’s kappa measured the level of agreement between the GeneXpert MTB/RIF result, radiologist and AI predictions.

Comparison	Cohen’s Kappa
GeneXpert vs Radiologist	0.69
GeneXpert vs AI Prediction	0.79
Radiologist vs AI Prediction	0.53

## Discussion

**Fig 3:**
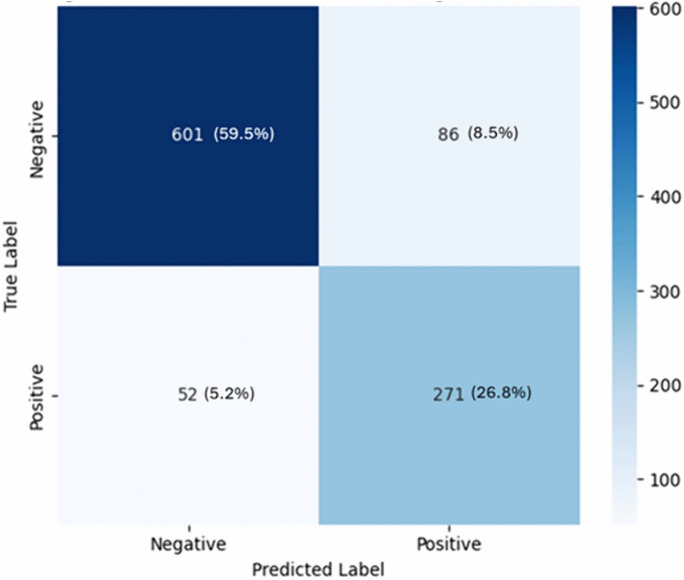
Confusion matrix for radiologist predictions.

**Fig 4:**
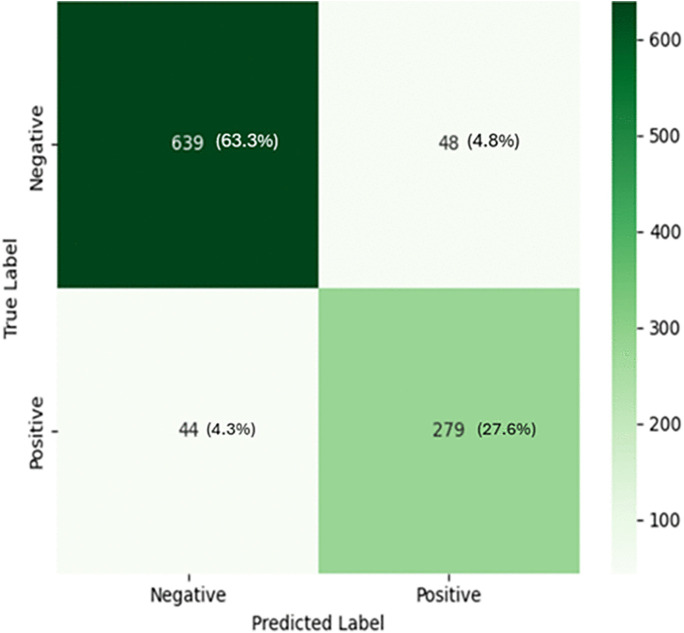
Confusion matrix for AI predictions.

**Fig 5:**
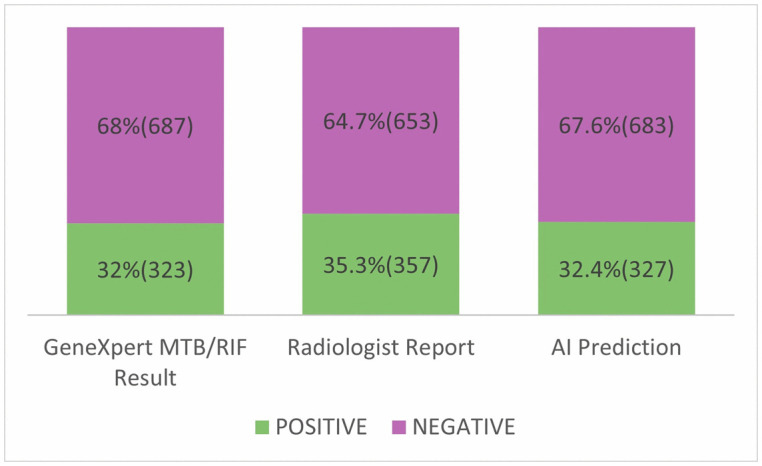
Distribution of TB cases across the three modalities.

This study evaluated the diagnostic performance of a radiologist and an AI-based system in screening for tuberculosis (TB) using a dataset of 1,010 patients. The results demonstrate that the AI system is comparable to the radiologist report across multiple performance metrics (Figs 2–5), including accuracy, sensitivity, specificity, PPV, NPV, and Cohen’s kappa agreement with the GeneXpert MTB/RIF reference standard. The AI system achieved an accuracy of 91%, sensitivity of 86%, and specificity of 93%, compared to the radiologist’s accuracy of 86%, sensitivity of 84%, and specificity of 87%. Also, Cohen’s kappa was used to measure inter-rater agreement. The agreement between the AI prediction and GeneXpert MTB/RIF result was substantial (κ = 0.79), higher than that of the radiologist (κ = 0.69). The moderate agreement (κ = 0.53) between the radiologist and AI predictions suggests that the two modalities may differ in their diagnostic approach, with the AI system potentially offering complementary insights. The significant result from McNemar’s test (p = 0.0021) further indicates that the AI system’s performance is not equivalent to that of the radiologist and may represent a meaningful improvement in TB screening accuracy. The ROC curves illustrate each modality’s ability to distinguish TB-positive from TB-negative cases. The AI system achieved a higher area under the curve (AUC = 0.90) compared with the radiologist (AUC = 0.86), indicating superior classification accuracy in distinguishing TB-positive from TB-negative cases as depicted in Fig 2. Furthermore, AI system’s false positive rate of 4.3% (Fig 3) is lower than that of radiologist with a rate of 8.5% (Fig 4).

The higher PPV and NPV values further support the AI’s robustness in clinical decision-making, particularly in settings where minimizing false positives and false negatives is critical. By serving as a triage tool, it can flag suspicious cases for prompt review, thereby reducing diagnostic delays and optimizing workflow efficiency at areas where there is a limited number of radiologist and or high patient volumes. Although the observed difference in accuracy between the two approaches is significant, its clinical relevance within Ghanaian tuberculosis screening programmes requires careful consideration. Laboratory testing in this study identified 32% TB-positive cases, while both the radiologist (35.3%) and AI (32.4%) predicted slightly higher proportions, with the radiologist reporting the highest (Fig 5). While the improvements in accuracy and specificity suggest a potential reduction in false-positive diagnoses (Fig 4) and unnecessary follow-up investigations, the modest gain in sensitivity may result in only a limited reduction in missed TB cases. In a high-burden setting such as Ghana, sensitivity is particularly critical as false-negative results can delay treatment initiation and contribute to ongoing transmission. Furthermore, the practical impact of these performance gains must be considered alongside contextual factors, including implementation costs of AI, infrastructure requirements, workforce capacity, and compatibility with existing radiology and TB control program workflows. Therefore, although the observed differences are statistically significant, their true clinical and public health value depends on whether the incremental improvements translate into meaningful enhancements in case detection, resource utilization, and overall TB control outcomes in Ghana.

These findings corroborate studies conducted in other regions of the world. Studies from across the globe consistently shows that AI systems for TB detection can perform at or above radiologist level, with variability largely attributable to reference standards, prevalence rates, and imaging protocols. To elaborate, in Zambia, CAD4TB was evaluated and reported a sensitivity of 88% and specificity of 75% against GeneXpert MTB/RIF, showing comparable performance to human readers in community screening [18]. The Western world documents AUC of above 0.90 whiles in Asia, AI AUC values fell between 0.951 and 0.975 with sensitivity and specificity above 85% [10,19,20]. These findings mirror our study outcomes, reinforcing the reliability and generalizability of AI models when locally calibrated. This study contributes to this global literature by demonstrating measurable improvements in diagnostic accuracy in Ghana, highlighting the potential impact of AI deployment in similar resource-limited settings.

These results underscore the feasibility of deploying AI-assisted radiography in Ghana. The integration of AI into radiographic workflows offers significant advantages in resource-limited settings, limited radiologist and high patient volumes can delay diagnosis [21]. AI-assisted interpretation can serve as a preliminary screening tool, flagging suspicious cases for further review and potentially accelerating the diagnostic process [22]. Moreover, the use of AI may help standardize interpretations across institutions, improving diagnostic equity and reducing the burden on stressed healthcare professionals. This is particularly relevant in rural and underserved areas where access to expert radiologists is very limited [23,24]. The tool’s offline functionality and rapid image processing make it particularly suitable for decentralized screening programs, mobile clinics and community healthcare initiatives [25].

However, several considerations must be addressed before widespread implementation. First, algorithmic performance may vary based on image acquisition protocols, patient demographics and disease prevalence [26–28]. Local calibration and continuous validation are essential to improve and ensure sustained accuracy and reduce algorithm bias [26]. Also, integration into existing health system requires training, infrastructure support and regulatory oversight to safeguard patient data and diagnostic integrity [29].

## Implications

In resource-limited settings, where access to expert radiologists exists, AI systems can serve as effective triage tools, reducing diagnostic delays and improving patient outcomes. The AI can augment clinical expertise by flagging high risk images for radiologist expediated review and laboratory confirmation.

## Limitations

The dataset of 1,010 patients with specific image requirement were derived from a single institution raising the issue of dataset and selection bias. This limits generalizability across different populations, imaging equipment, and clinical contexts (both rural and urban cultural settings) within the country. Secondly, GeneXpert MTB/RIF results derived from clinical files were used as the reference standard. Although this test is sensitive and specific, it does not capture all cases of active tuberculosis, and misclassifications inherent in the test may have influenced performance estimates. Additionally, the CNN was fine-tuned using publicly available TB chest X-ray datasets some of which were from the Ghanaian setting, however this is still dilute and lacks full representation of Ghanaian patients’ demographics, radiographic patterns, comorbidities, body habitus, skin tones, and imaging variability, potentially causing algorithmic bias, domain shift, and performance degradation in local contexts.

Finally, performance metrics such as positive and negative predictive values are influenced by disease prevalence, and may not directly translate to settings with different epidemiological profiles. These limitations underscore the need for multicenter validation studies, incorporation of diverse patient populations and cultures with continuous local calibration specific to the Ghanaian population to ensure sustained accuracy and equitable deployment of AI-assisted screening tools.

## Conclusion

AI-assisted chest radiograph interpretation offers a promising solution to enhance early TB screening in resource-limited settings like Ghana. Its high diagnostic accuracy, scalability, and consistency position’s it as a valuable tool in the fight against TB. Strategic implementation, supported by local validation and policy frameworks, could significantly improve early detection and reduce TB-related morbidity and mortality.

## Supporting information

S1 Data Supplemetary file data. (CSV)
